# The dual topoisomerase inhibitor A35 preferentially and specially targets topoisomerase 2α by enhancing pre-strand and post-strand cleavage and inhibiting DNA religation

**DOI:** 10.18632/oncotarget.5680

**Published:** 2015-10-07

**Authors:** Wuli Zhao, Guohua Jiang, Chongwen Bi, Yangbiao Li, Jingbo Liu, Cheng Ye, Hongwei He, Liang Li, Danqing Song, Rongguang Shao

**Affiliations:** ^1^ Key Laboratory of Antibiotic Bioengineering, Ministry of Health, Laboratory of Oncology, Institute of Medicinal Biotechnology, Peking Union Medical College and Chinese Academy of Medical Sciences, Beijing, China; ^2^ Analysis and Testing Center, Beijing Normal University, Beijing, China; ^3^ China Meitan General Hospital, Beijing, China

**Keywords:** dual topoisomerase inhibitor, topoisomerase2α, cardiotoxicity, DNA religation, enhancing strand cleavage

## Abstract

DNA topoisomerases play a key role in tumor proliferation. Chemotherapeutics targeting topoisomerases have been widely used in clinical oncology, but resistance and side effects, particularly cardiotoxicity, usually limit their application. Clinical data show that a decrease in topoisomerase (top) levels is the primary factor responsible for resistance, but in cells there is compensatory effect between the levels of top1 and top2α. Here, we validated cyclizing-berberine A35, which is a dual top inhibitor and preferentially targets top2α. The impact on the top2α catalytic cycle indicated that A35 could intercalate into DNA but did not interfere with DNA-top binding and top2α ATPase activity. A35 could facilitate DNA-top2α cleavage complex formation by enhancing pre-strand and post-strand cleavage and inhibiting religation, suggesting this compound can be a topoisomerase poison and had a district mechanism from other topoisomerase inhibitors. TARDIS and comet assays showed that A35 could induce cell DNA breakage and DNA-top complexes but had no effect on the cardiac toxicity inducer top2β. Silencing top1 augmented DNA break and silencing top2α decreased DNA break. Further validation in H9c2 cardiac cells showed A35 did not disturb cell proliferation and mitochondrial membrane potency. Additionally, an assay with nude mice further demonstrated A35 did not damage the heart. Our work identifies A35 as a novel skeleton compound dually inhibits topoisomerases, and predominantly and specially targets top2α by interfering with all cleavage steps and its no cardiac toxicity was verified by cardiac cells and mice heart. A35 could be a novel and effective targeting topoisomerase agent.

## INTRODUCTION

DNA topoisomerases are essential enzymes for cells to modulate DNA topology by regulating the over- or under-winding of DNA strands during cellular processes such as DNA transcription, replication, or recombination [1]. Top1 is a nuclear enzyme that catalyzes the relaxation of superhelical DNA by generating a transient single strand nick. Top2 mediates the ATP-dependent induction of coordinated nicks in both strands of the DNA duplex, followed by the crossing of another double strand of DNA through the transiently broken duplex. Two top2 isozymes are expressed in humans: top2α and top2β. Top2α is most abundantly expressed in rapidly growing tissues, and its expression is cell cycle-regulated, peaking during G2/M. In contrast, top2β is ubiquitously expressed in terminally differentiated cells including cardiomyocytes and its expression levels do not exhibit any significant changes during the cell cycle [2–5].

Given that they are highly expressed in aggressive cancer cells and are essential to cancer cell survival, top1 and top2α are potential drug targets for treating human malignancies. Compared with top1, top2α is more essential for cell viability because only top2α can drive the separation of two DNA duplexes after replication and its deletion is lethal for cell survival [6–8]. Chemotherapeutics targeting top1 and in particular top2α have been of great utility in clinical oncology. Although these drugs are highly effective, tumors frequently recur and even become resistant, and the occurrence of side effects, particularly cardiotoxicity and secondary malignancies, tremendously limit their application. Some studies have shown that decreased topoisomerase levels in relapsed tumors contribute to the tumor resistance [9, 10]; additionally, researchers also found that there is a compensatory effect between top1 and top2α: when the expression of one type of topisomerase is decreased, the other will be increased [9, 11–13]. Some evidence regarding the adverse effects, particularly anthracycline-induced cardiotoxicity mediated by targeting top2 indicated that non-specific targeting of top2β is the initial molecular mechanism underlying this phenomenon [14]. Thus, to overcome the above-mentioned drug resistance and cardiotoxicity, combinatorial targeting of top1 and top2α is essential, but recent clinical data have shown that the combination of top1 and top2α poisons might lead to severe life-threatening neutropenia and anemia resulting from the toxicity overlay of the two agents [13]. Thus, it is extremely important to identify a compound that simultaneously targets top1 and top2α. Although some researchers have investigated compounds that could target top1 and top2, they did not precisely elucidate whether the top2 target is top2α or top2β [15–18]. Recently, one study in the literature reported an agent that could inhibit top1 and top2α, but it did not further clarify its effects on top2β [19]. Additionally, the predicted lack of cardiac toxicity of reported inhibitors specifically targeting top2α was obtained only based on a cell-free assay (DNA directly incubated with synthetic topoisomerase in buffer); further validation in cardiac cells and drugs effects on animal cardiac muscle were not reported [20–23].

**Figure 1:**
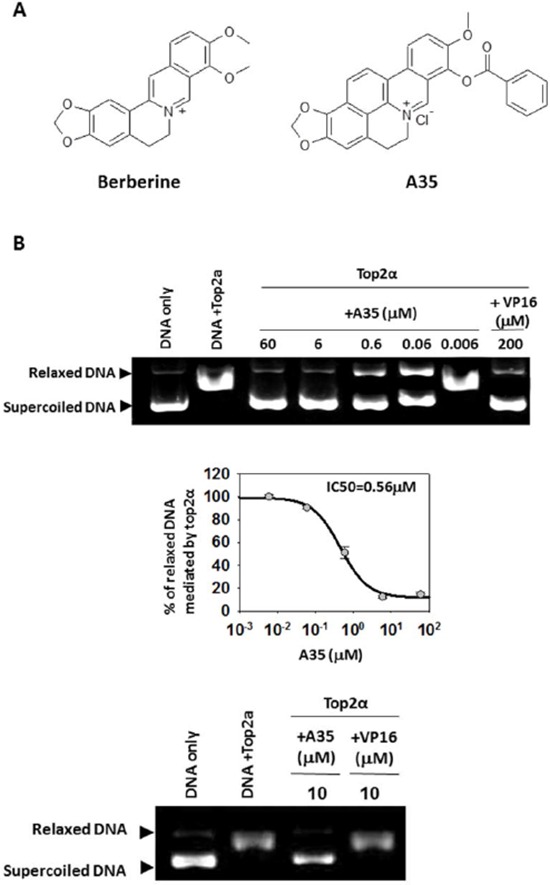

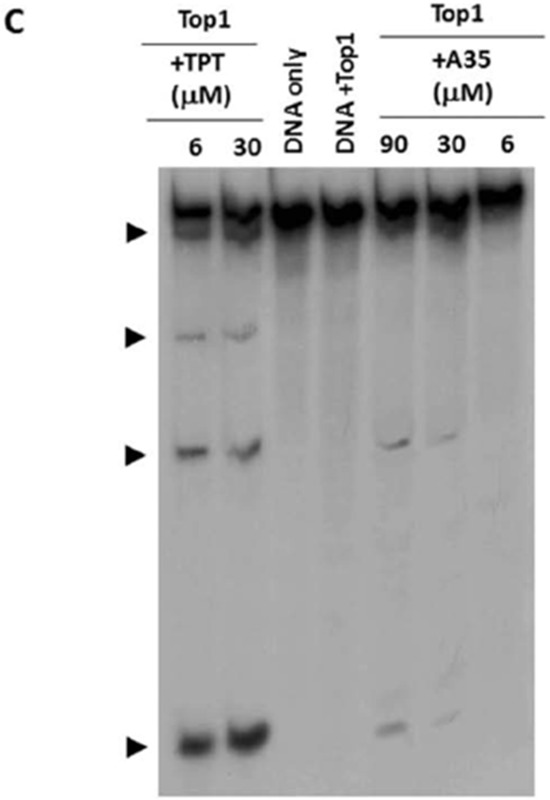
A35 is a dual inhibitor of top2α and top1 **A.** The structure of Berberine and A35. **B.** 0.5 μg supercoiled plasmid pBR322DNA was incubated with top2α and various concentrations of A35 or the indicated agents for 30 min. The reaction was stopped and the reaction products were separated in 1% agarose. RLX, relaxed DNA; SC, supercoiled DNA. The relaxed DNA was scanned by Image J and the IC50 was defined as the concentration of A35 resulting in a 50% reduction of relaxed DNA. Data represent the mean ± S.E.M. of three independent experiments. **C.** 3′-end-labeled 117-bp oligonucleotide was reacted with top1 (60U) in the presence or absence of indicated concentrations of compound. Arrowheads indicate the migration positions of DNA fragments cleaved by top1 in the presence of compounds.

Berberine (BBR, Figure 1A, left), an isoquinoline natural product extracted from Coptis chinensis, has been extensively used as an anti-inflammatory [24], cholesterol-lowering [25] and antineoplastic [26] research agent. However, its anticancer activity is weak [26, 27]. Cyclizing-berberine is a novel skeleton compound (berberine of 1, 13-cyclication) that is occasionally obtained during the structural transformation of berberine in the search for a highly effective cholesterol-lowering agent. A screen found that this class of compound could inhibit cell proliferation; this detection evoked our interest to determine whether this novel structural class compound induced anticancer activity.

Via successive structural transformation (A series of new cyclizing-berberine derivatives were synthesized through variations at the 9-position) and repetitive cytotoxicity assays, cyclizing-berberine A35 (replacement of 9-methoxyl with an ester moiety) (Figure 1A, right) emerged and contained a greater number of aromatic rings that facilitate intercalation into DNA or topoisomerase, and also displayed excellent anticancer activity that was clearly better than its parent berberine (data not shown). A toxicity assay further confirmed its lower toxicity (300 mg/kg administration by intraperitoneal injection; the mouse survival rate was 100%).

In the present study, we evaluated the novel skeleton compound A35 in a cell-free assay, a cell assay and in further animal experiments and demonstrated that A35 could target top1 and particularly top2α by increasing pre-strand and post-strand cleavage and inhibiting the religation. Additionally, we also illustrated that A35 could specifically target top2α and not top2β; meanwhile, we confirmed that A35 did not induce cardiac cell injury in rat cardiomyocyte cells and nude mouse hearts.

## RESULTS

### A35 is a dual inhibitor of top2α and top1

Given that A35 possesses a greater number of aromatic rings and its structure is similar to known topoisomerase2 inhibitors [22, 23], we first examined the effects of A35 on top2α activity by top2α-mediated relaxation assay. As shown in Figure 1B, A35 significantly inhibited top2α relaxation activity in a concentration-dependent manner. The relaxed DNA was quantified by software Image J and IC50 (a concentration resulting in 50% relaxed DNA reduction) was calculated as 0.56 μM by SigmaPlot. At the same concentration (10 μM), inhibitory activity of A35 on top top2α was much higher than that of etoposide (VP16), indicating that A35 was a powerful top2α inhibitor. Then, we utilized top1-mediated cleavage assay with linear DNA as substrate to evaluate the effect of A35 on top1, and results showed that A35 could induce linear DNA breakage in dose-dependent manner (Figure 1C), although the DNA breakage effect was weak and far less than the positive control TPT (topotecan), a water-soluble derivative of alkaloid camptoth ecin (CPT) and could stabilize top1 cleavage complexes [28, 29], indicating A35 could inhibit top1 activity. Similarly top1-mediated relaxation assay showed that the IC50 of A35 on top1 was 22.1 μM that was far higher than the IC50 of A35 on top2α (data not shown). These results showed that A35 could dually inhibit top2α and top1, although the effect on top2α was be superior to the effect on top1.

### A35 can intercalate DNA but does not interfere with top2α-DNA binding and top2α ATPase activity

**Figure 2:**
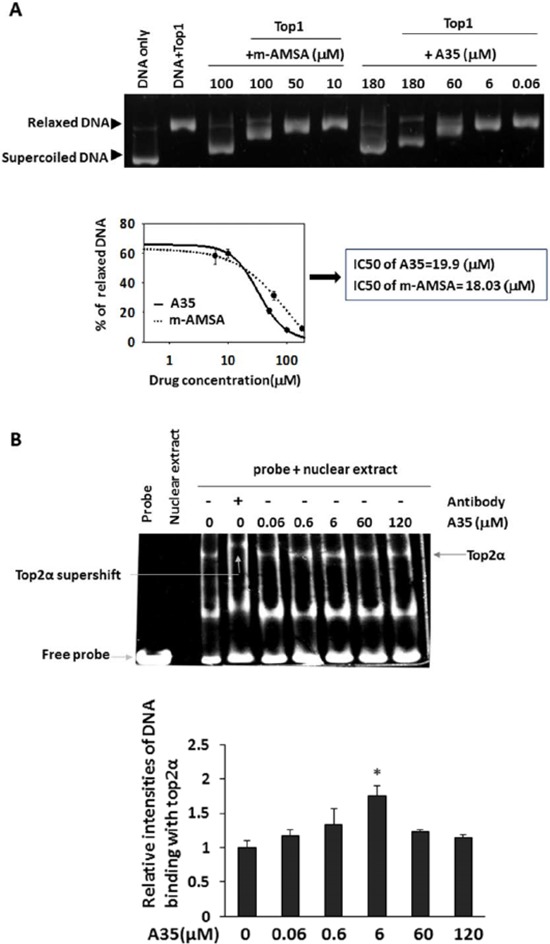

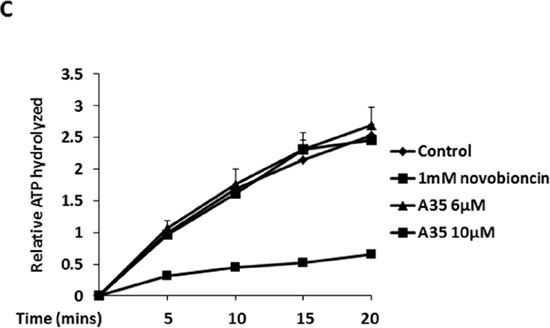
A35 can intercalate DNA but does not interfere with top2α-DNA binding and top2α ATPase activity **A.** Plasmid pBR322 DNA was firstly relaxed by top1 prior to the addition of the indicated concentration agents and incubated for a further 30 min at 37°C. Reaction products were resolved on an agarose gel prior to visualization with ethidium bromide. The relaxed DNA was scanned and the dose response curves and IC50 were plotted and depicted. The IC50 represents the concentration required to facilitate 50% relaxed DNA to form supercoiled DNA. **B.** K562 nuclear extracts were incubated with 1 nm double-stranded oligonucleotide containing a strong top2α binding site in the presence of increasing concentrations of A35. After supershifting, an antibody was first incubated with nuclear extracts, and then the DNA probe and compound were added. Reaction products were resolved by non-denaturing polyacrylamide gel electrophoresis, the intensities of band were scanned and the relative ratio to nuclear extract+probe was plotted. **C.** The effects of A35 on top2α hydrolysis activity were determined by thin layer chromatography with [γ32P]-ATP in the presence of indicated compounds. Samples from various time points were quantified by scintillation counting and plotted relative ratio to the 5 min scintillation counting of control.

Given that top2α is indispensable for cell division and was strongly inhibited by A35, in the following experiment we focused on investigating the effects of A35 on top2α. There are several steps in the top2α catalytic cycle. As a consequence, multiple independent approaches are required to determine the mechanisms underlying the drug-mediated inhibition of top2α. For A35, its intercalation into DNA is attributed to its polar structure, and this intercalation might lead to the topoisomerase DNA binding site being occupied or distortion of the DNA backbone; this can then interfere with top2-DNA binding. The ability of A35 to intercalate DNA was assessed using a top1 unwinding assay as described [20], pBR322DNA was firstly relaxed by top1 and mAMSA (amsacrine) (a known DNA intercalator) as a positive control [30] or A35 was added, then we observed the negative supercoiling formed after the addition of mAMSA or A35 (Figure 2A). The relaxed DNA was quantified, and the dose-response curve and IC50 (a concentration of 50% relaxed DNA reduction) showed almost identical DNA intercalation activity between A35 and mAMSA, indicating that A35 was a DNA intercalator.

Given that A35 could intercalate into DNA, we utilized an EMSA assay [31, 32] to measure whether A35 interfered with top2α-DNA binding (Figure 2B) as described. A DNA probe containing a robust top2α-binding site was synthesized and incubated with nuclear extracts; after electrophoresis, we observed the DNA-top2α binding complex at the position of the top2α protein in the gel, and further verification that the complex contained the top2α protein was obtained via supershift assay with the anti-top2α antibody. Additionally, we determined that A35 did not disturb DNA-top2α binding at various concentrations; interestingly we found that at 6 μM A35 seemingly promoted the binding of DNA and top2α slightly, and at the highest concentration of 120 μM the binding activity decreased compared with 6 μM.

The effect of A35 on top2α ATP enzyme activity was examined by thin layer chromatography with [γ-32P]-ATP and results showed that novobiocin (a known top2α ATP enzyme inhibitor) [33] significantly suppressed top2α-mediated ATP hydrolysis, demonstrating that the assay was credible. But in A35-treated groups, we did not observe significant changes of ATP hydrolysis mediated by top2α, indicating that A35 did not inhibit top2α ATP enzyme activity (Figure 2C).

### A35 facilitates top2α-DNA cleavage complex formation by simultaneously enhancing pre-strand and post-strand cleavage and inhibiting DNA religation

**Figure 3:**
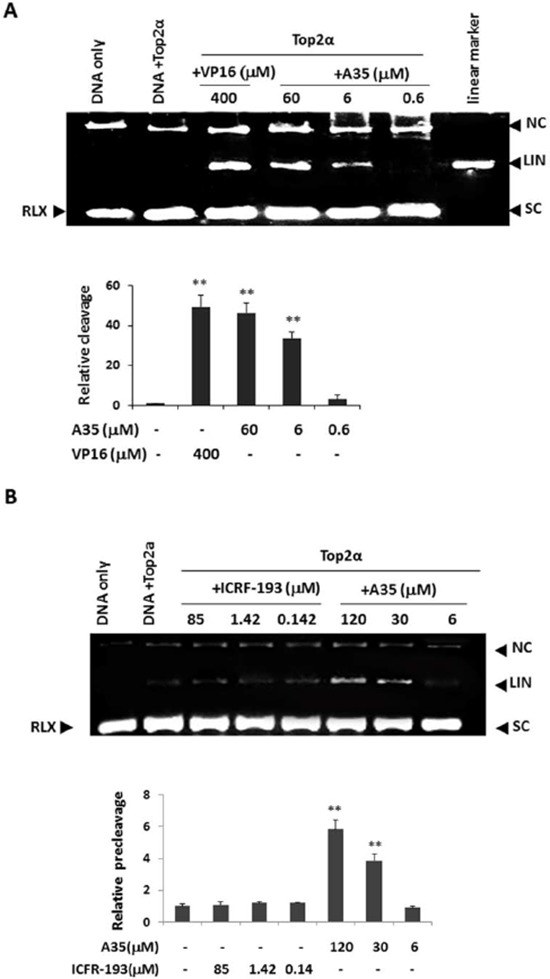

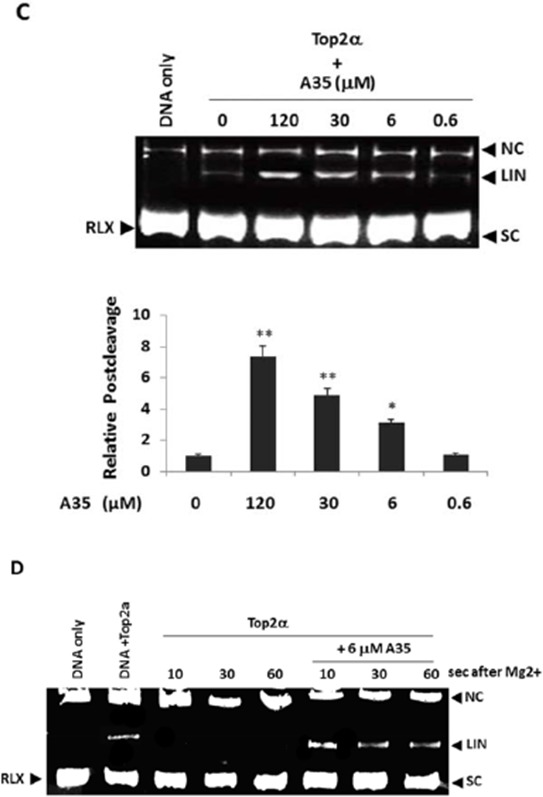
A35 induces top2α-DNA cleavage complex formation by enhancing pre-strand and post-strand cleavage and inhibiting DNA religation **A.** The supercoiled plasmid pBR322 was incubated with purified top2α (8U) with or without the indicated concentrations of A35. Reaction products were separated by 1.4% agarose gel electrophoresis in the presence of the nucleic staining agent EB to allow for the separation of supercoiled (SC), relaxed closed-circular (RLX), linear (LIN) and nicked circular (NC) DNA. The intensities of the linear bands observed were quantified and plotted relative to the control (pBR322+Top2α). **B.** To determine the effects of A35 on top2α-mediated pre-strand passage cleavage, reactions were performed in the absence of ATP, and the intensities of the linear bands were quantified and plotted relative to the control (pBR322DNA+Top2α). **C.** Top2α-mediated post-strand passage DNA cleavage affected by A35 was carried out in a reaction buffer containing AMPPNP (1 mM) instead of ATP, and the resulting graph was constructed. **D.** Top2α-mediated religation of the pBR322 plasmid was examined in the presence or absence of A35. Kinetically competent top2-DNA complexes were trapped in the presence of Ca^2+^ and in the absence of ATP. After the addition of A35, reactions were reinitiated with Mg^2+^ and trapped at the indicated time points and examined. *P < 0.05;** *P* < 0.01

Then we evaluated the effect of A35 on the DNA cleavage. A cleavage assay in the presence of 8 U top2α, 0.2 μg pBR322 and increasing concentrations of A35 was performed, and the results showed that cleaved linear DNA was formed and the cleaved band presented in a dose-dependent manner (Figure 3A). Together, these results indicated that A35 stabilized the DNA-enzyme complex and thus this compound belongs to the poison of topoisomerase inhibitors.

Usually pre-strand cleavage and post-strand cleavage participated in the top2α-mediated DNA cleavage process [20]. Interference with any step would induce the occurrence of DNA cleavage and linear DNA formation. As ATP is required for strand passage, in the absence of ATP top2 binds DNA and establishes prestrand cleavage-religation equilibrium prior to strand passage. As seen in Figure 3B, a significant increase in the amount of cleaved linear DNA was observed by addition of A35 (120 μM and 30 μM) and the cleaved band presented in a dose-dependent manner, indicating that A35 enhanced pre-strand cleavage reaction. Just in accordance with previous description [20], meso-4,4′-(3,2-butanediyl)-bis(2,6-piperazinedione) (ICRF-193) inhibited enzyme activity at the post-cleavage and strand passage steps, had no effects on the formation of cleaved DNA or on the levels of nicked circular DNA in the pre-cleavage stage as a negative control.

To determine if A35 has similar effects on post-strand passage DNA cleavage, we repeated the above experiment, but with the addition of Adenylyl-imidodiphosphate(AMPPNP) in the reaction buffer [34]. The addition of this non-hydrolyzable ATP analog permits strand passage to occur. In the presence of AMPPNP and Mg^2+^, incubated with increasing concentrations of A35 led to cleaved DNA increased (Figure 3C), and indicated that A35 also prompted post-strand cleavage complex formation.

We subsequently examined the effects of A35 on top2α-mediated DNA religation. We kinetically trapped top2α-DNA complexes in the presence of Ca^2+^. After using EDTA to chelate the excess Ca^2+^ ions, Mg^2+^ reintroduction triggered the religation of the linear band [20]. To examine whether A35 impacts the religation of DNA by top2α, we initiated reactions with Mg^2+^ in the presence or absence of A35 (Figure 3D). The experiments demonstrated that the religation of DNA occurred almost immediately and was severely hindered by the addition of A35. Together, these results indicated that A35 could enhance top2α-mediated pre-strand and post-strand DNA cleavage and inhibit DNA religation.

### Compared with top1, top2α is preferentially and specifically targeted in A35-induced cell DNA strand breaks, top-DNA covalent complexes and growth inhibition

**Figure 4:**
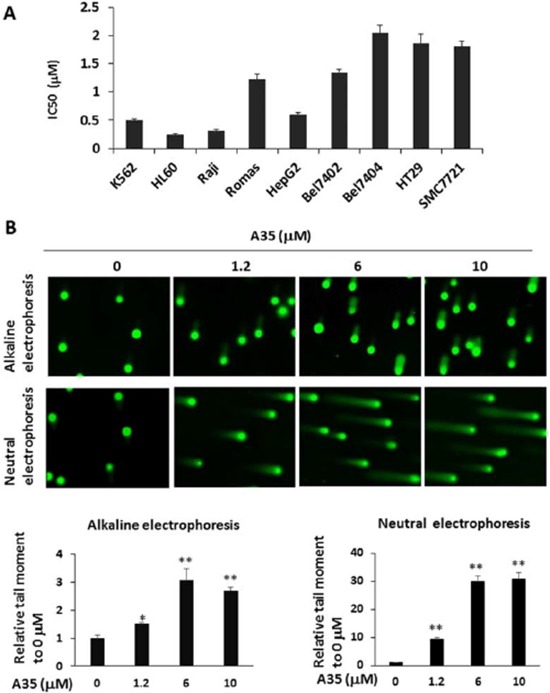

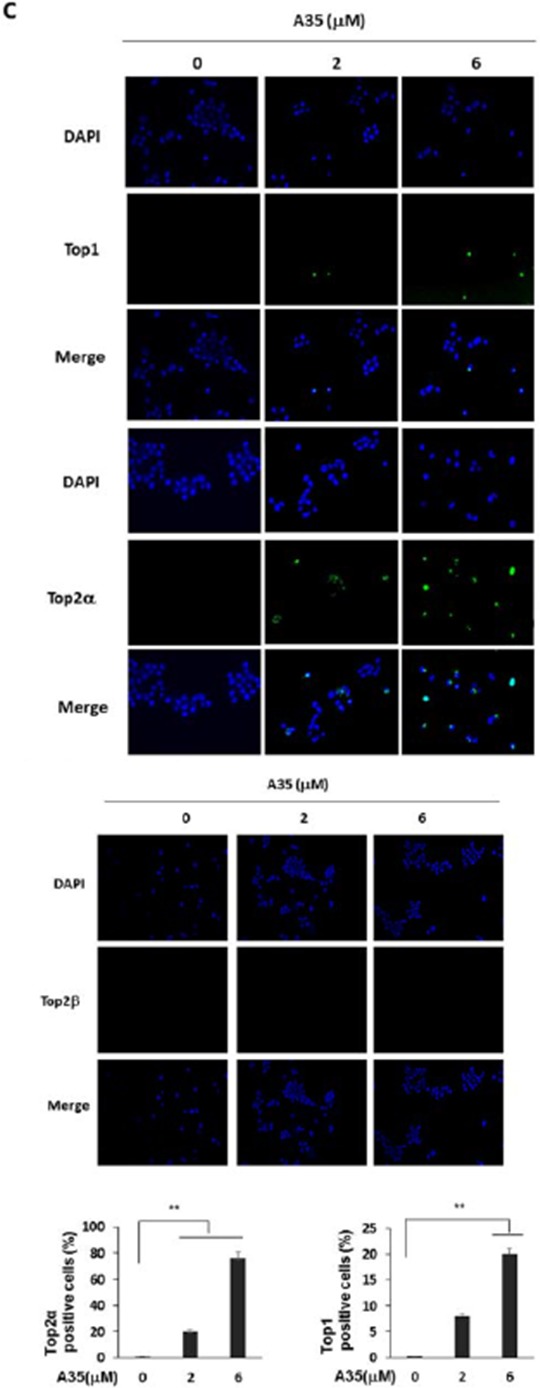

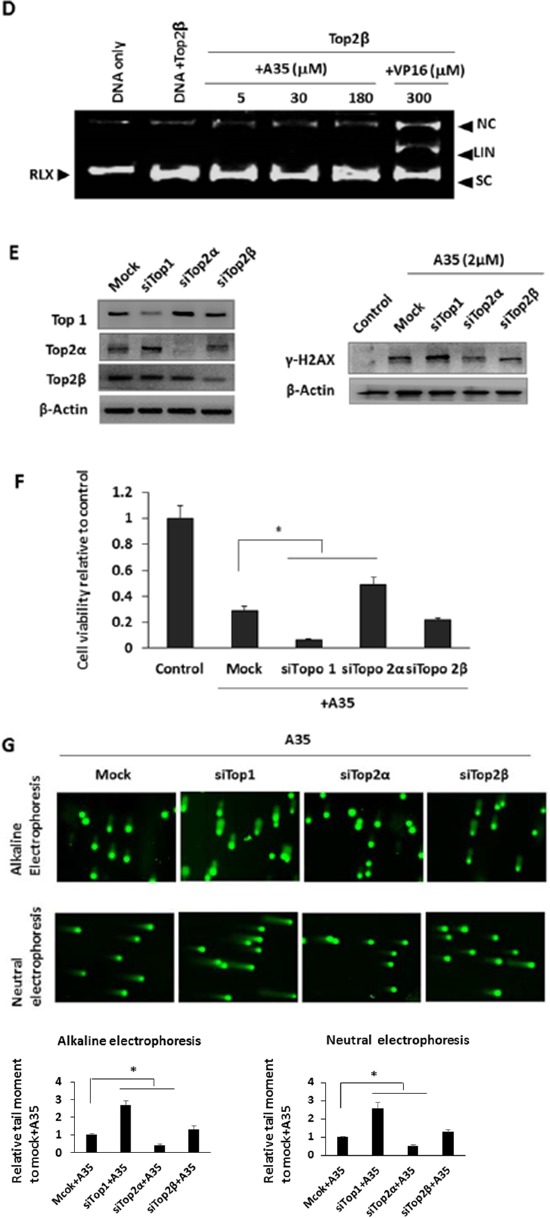
Compared with top1, top2α was preferentially and specifically targeted in A35-induced cell DNA strand breaks, top-DNA covalent complexes and growth inhibition **A.** IC50 of A35 in various cancer cells. Cells were treated with different concentrations of A35 for 48 hrs, and IC50 was evaluated with SigmaPlot. **B.** K562 cells were treated for 1.5 hours in the presence of A35, and cells were collected and alkaline comet electrophoresis and neutral comet electrophoresis assays were performed. The quantitative analysis was performed with the comet analysis software CASP, and the parameters TM (tail moment) was employed to evaluate DNA damage. Assays were repeated three times and the mean value of TM was normalized to the control (0 μM). **C.** K562 cells were treated with increasing concentrations of A35 for 1.5 hours, and the formation of DNA-top covalent complexes was analyzed by TARIDS assay. Nuclei were counterstained with DAPI. The histogram shows the percentage of top2α-positive or top1-positive cells. The results of a single experiment are shown; 50 nuclei were analyzed in each assay. **D.** The DNA pBR322 was incubated with purified top2β with or without the indicated concentrations of A35 or VP16. Reaction products were separated by 1.4% agarose gel electrophoresis in the presence of the nucleic staining agent EB. K562 cells were transfected with top1 or top2 siRNA for 48 hours, and top1 and top2 levels were detected **E.** 1.2 μM A35 was then added into the top1 or top2 silenced cells and incubated for 24 hours to examine γ-H2AX levels (E) and cell survival **F.** Incubation for 1.5 hours to detect DNA single or double strand breakages via alkaline or neutral comet electrophoresis **G.** *P < 0.05;** *P* < 0.01

Although the cell-free assay showed that A35 was a dual inhibitor against top1 and top2α, true target identification should be obtained in a cell assay. First, we used K562, HL60, Raji and Romas hematological tumor cells as well as solid tumor cells including the hepatic carcinoma cell lines HepG2, Bel7402 and Bel7404 and colorectal cancer cell lines to evaluate the growth inhibitory effects of A35. The results showed that the IC50 for all cancer cells except for Bel7404 was less than 2 μM, indicating good proliferation inhibitory activity (Figure 4A). Given that the cell-free assay showed that A35 induce top1 and top2α mediated DNA single or double DNA cleavage, we examined DNA single strand breakage with an alkaline comet assay [35] and double strand breakage with a neutral comet assay in cells treated with A35. The results showed that A35 could induce single strand DNA breakage, and at 1.2 μM the tail moment (TM) was about 1.5 folds of control, and at higher concentrations (6 μM and 10 μM) the tail moments were approximately 3-fold of the control (Figure 4B, upper). Neutral comet electrophoresis indicated that A35 could induce obvious DNA double breakage at each concentration and in a dose-dependent manner: at the lowest concentration of 1.2 μM, the tail moment was approximately 9-fold of the control, and at 6 μM and 10 μM the tail moments were up to 30-fold of the control, indicating that A35 induced more double strand breakage (Figure 4B, lower).

The cell-free assays showed that A35 could induce DNA-top1 or -top2α complex formation. We then utilized the TARDIS (trapped in agarose-DNA immunostaining) assay to further clarify the effects in cells. As shown in Figure 4C, top1-DNA and top2α-DNA complexes were observed in a dose-dependent manner after treatment with A35, and at 6 μM the top2α-positive cells were up to 80% and top1-positive cells were approximately 20% of the total. However, we did not visualize the top2β-DNA complex at any concentration of A35. To further ascertain top2β is not the target of A35, we performed top2β-mediated DNA cleavage assay in the presence of A35 to evaluate the effect of A35 on DNA cleavage, and results also demonstrated that A35 did not lead to DNA breakage mediated by top2β and the positive (VP16) control induced significant DNA breakage (Figure 4D), indicating A35 did not target top2β.

To verify top1 and top2α are the primary target of A35 to induce DNA breakage followed by cell death, we knocked down top1, top2α and top2β and further assessed whether A35-induced cell proliferation inhibition and DNA breakage that was lethal to cell survival could be reversed,. As shown in Figure 4E, the topoisomerases were knocked down, and we found there was a compensatory effect between top1 and top2α, specifically that when top1 decreased top2α would increase and vice-versa. However, in the top2β-silenced cells, there were only minor changes in top2α and top1 levels. To these topoisomerase knockdown cells, we added 2 μM A35 for 24 hours and evaluated levels of the double-strand break (DSB) damage marker γ-H2AX. The results showed that γ-H2AX levels increased in top1 knockdown cells and decreased in top2α knockdown cells, but obvious changes were not detected in top2β knockdown cells (Figure 4E). Then, we examined the effects of A35 on cell proliferation after topoisomerase silencing, and the results showed that with the knockdown of top1, the proliferation inhibitory activity induced by A35 was strengthened but was reversed after top2α was silenced, and there was no significant change in top2β-silenced cells (Figure 4F). The alkaline and neutral comet assays also showed that in top1-silenced cells, single and double strand DNA breakage all increased, while in top2α-silenced cells single and double strand DNA breakage decreased (Figure 4G). These results indicated that top1 and top2α were all targets of A35, but top2α was a more vital target and A35 did not target top2β.

### A35 does not induce cardiac cell cytotoxicity and mitochondrial damage and induces cancer cell apoptosis but not through the mitochondrial pathway

**Figure 5:**
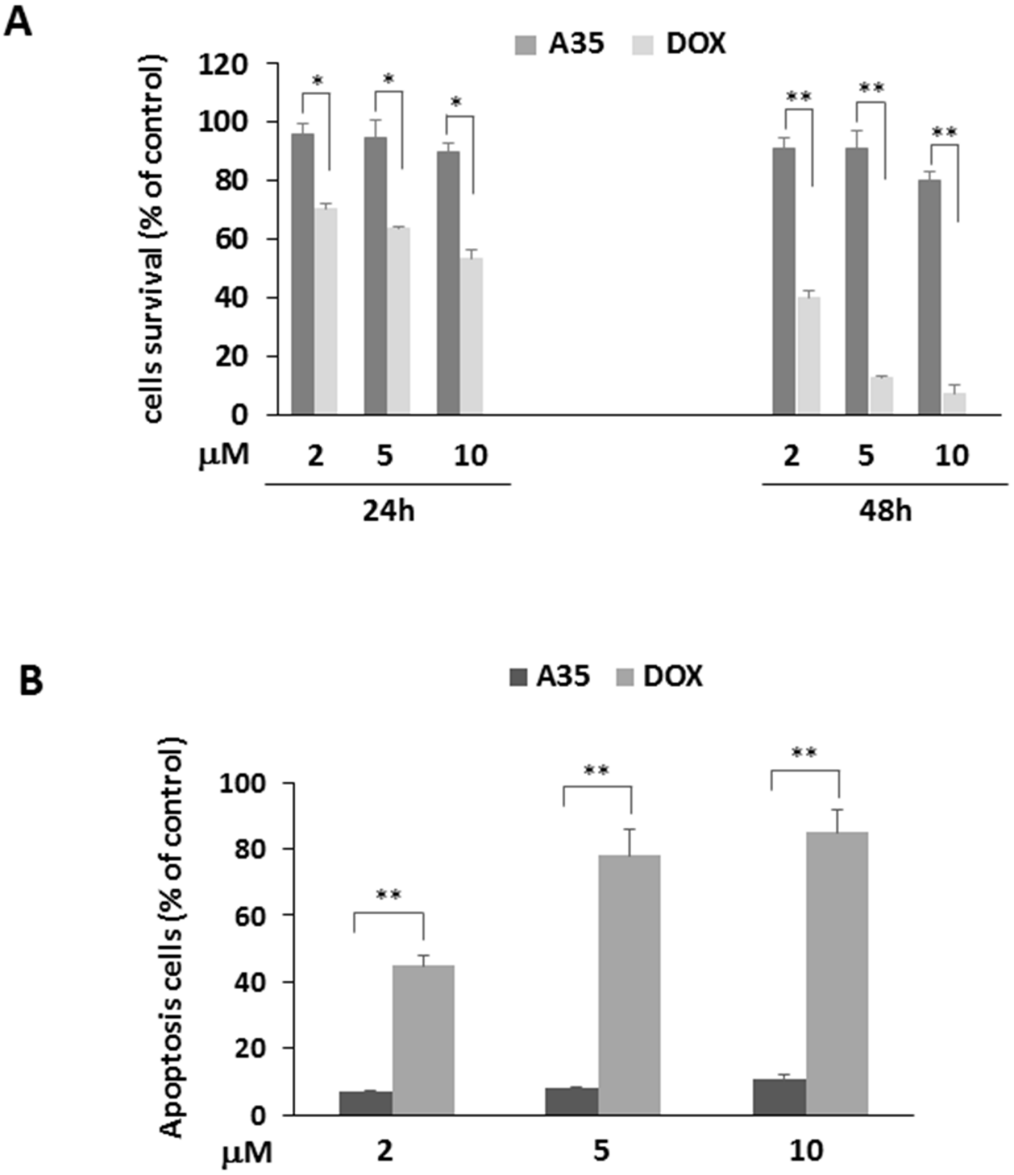

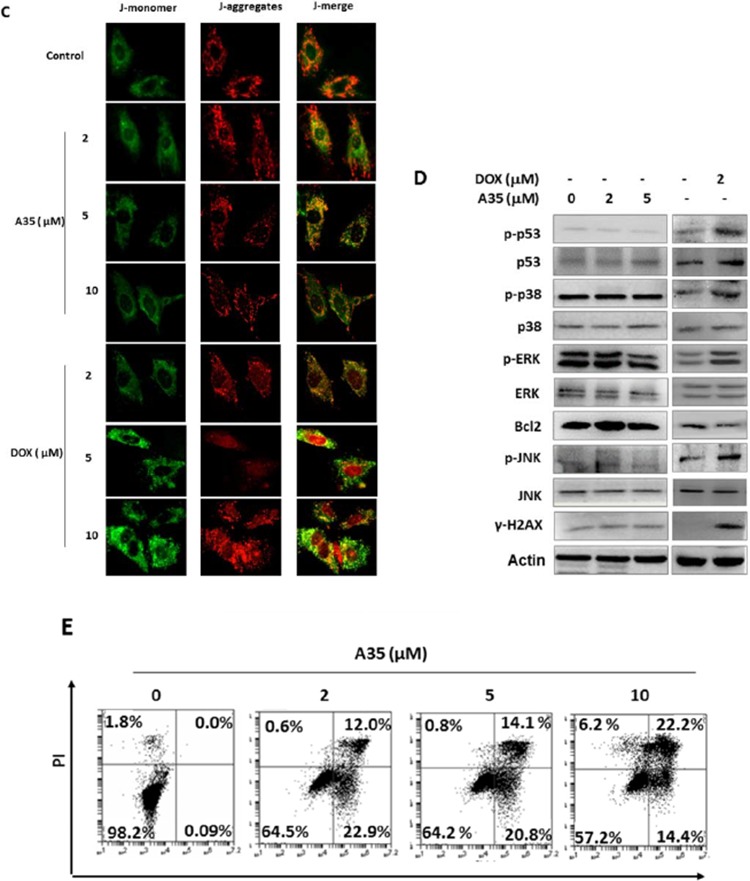

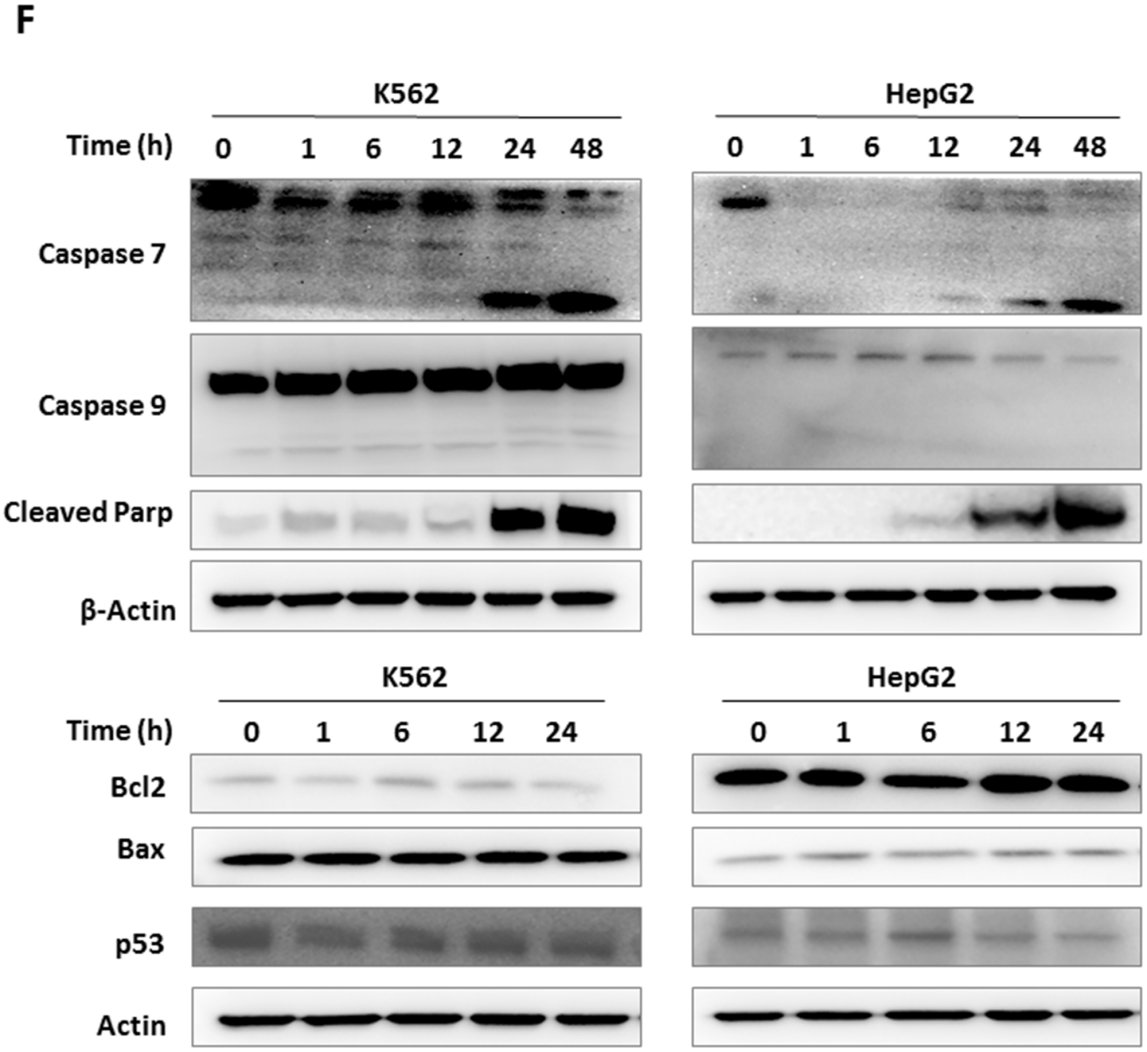
A35 does not induce cardiac cell cytotoxicity and mitochondrial damage and induces cancer cell apoptosis but not through the mitochondrial pathway H9C2 cells were treated with 2 μM, 5 μM and 10 μM A35 or DOX for 24 or 48 hours, cell viability was detected by MTS **A.** and apoptosis was detected by Annexin V-FITC and PI-staining after 48 hours of treatment and the histogram was constructed and presented **B. C.** H9C2 cells plated on coverslips were maintained in medium with or without 2 μM, 5 μM and 10 μM A35 or DOX for 24 h. Cells were stained with JC-1 and examined under fluorescence microscopy for red JC-1 aggregates or green JC-1 monomers, as described in the Materials and Methods. Typical images are shown. **D.** H9C2 cells were treated with indicated concentrations of A35 or DOX for 24 hours and mitochondrial damage-associated proteins or DNA damage proteins were measured by western blot. **E.** K562 cells were treated with increasing concentrations of A35 for 48 hours, and apoptosis was determined by flow cytometric analysis of Annexin V-FITC and PI-staining. **F.** K562 cells were treated by A35 and collected at the indicated time points for western blot analysis of apoptosis-associated proteins controlled by the mitochondria. *P < 0.05;** *P* < 0.01 compared with DOX.

Currently, topoisomerase 2 inhibitors are effective antineoplastic agents and have been widely used in tumor therapy. However, given their adverse effects, the application of topoisomerase 2 inhibitors, especially anthracyclines such as doxorubicin (DOX), has been restricted primarily due to the serious cardiac toxicity that results from targeting top2β. Although the above cell-free and cell-based assays verified that A35 could not target top2β, for further verifying the non-cardiotoxic effects of A35 we utilized H9C2 cardiac myoblasts from rats to perform the following assays. First, we evaluated whether A35 exerted proliferation inhibitory effects on H9C2 cells, and the results showed that after 24 h treatment with A35, at the concentrations of 2 μM and 5 μM cell vitality was almost equal to the control, and at 10 μM cell survival was approximately 90% of the control. After treatment for 48 h, H9C2 cell survival was approximately 90% of the control at 2 μM and 5 μM and 80% at 10 μM (Figure 5A), indicating that A35 barely interfered with cardiac cell proliferation. However, in the doxorubicin-treated group, we visualized obvious cardiac cell growth inhibitory effects after 24 h of treatment, and at 48 h, most cells were dead at higher concentrations. Specifically, after 24 h treatment cell survival was 70.2% of the control at 2 μM, 63.5% at 5 μM and 53.2% at 10 μM, and after 48 h treatment cell survival was 40.3% at 2 μM, 12.5% at 5 μM and 7.3% at 10 μM. Similarly, after the addition of DOX for 48 h, obvious apoptosis occurred, and the apoptotic cells were approximately 45% at 2 μM, 78% at 5 μM and 85% at 10 μM, but there was only 7–12% apoptotic cells observed in the A35-treated group (Figure 5B).

A number of studies have suggested that mitochondrial dysfunction is the primary molecular mechanism underlying DOX-induced cardiotoxicity. Alterations in mitochondrial membrane potential were the main effects of DOX in the mitochondria. Recent studies showed that DOX-induced mitochondria dysfunction is entirely attributable to the targeting of mitochondria top2β, leading to mitochondrial nucleic acid synthesis blockage and causing the collapse of membrane potential, finally leading to whole-cell energy supply obstruction and cell death [14, 36]. We detected the effects of A35 on mitochondrial membrane potential with JC-1. JC-1 is a fluorescent dye that exhibits potential-dependent accumulation in the mitochondria and can selectively enter the mitochondria as J-aggregates (JC-1 aggregates) with intense red-orange fluorescence in normal cells. If the membrane potential is disturbed, the dye remains in the monomeric form as J-monomer, emitting only green fluorescence. In our experiments, shown in Figure 5C, red granular aggregates were observed in the cytoplasm after excitation at 488 nm, both in untreated H9C2 cells or cells treated with various concentrations of A35, indicating that A35 did not alter the mitochondrial membrane potential of H9C2 cells. However, in doxorubicin-treated cells, at 2 μM red granular aggregates obviously decreased in the cytoplasm, and at higher concentrations (5 μM and 10 μM), red fluorescence aggregates almost entirely disappeared, indicating that cell mitochondria membrane potency was dramatically destroyed. Given that top2 was mainly located in the nucleus and doxorubicin itself emits red fluorescence, we observed red fluorescence in cells, particularly in the nuclei. Meanwhile, in the doxorubicin-treated group, we observed cytoplasm that was distinctly wrinkled, distorted and broken. In comparison, the cytoplasm in A35-treated H9C2 cells was plump both at low or high concentrations, indicating that A35 did not injure cardiac cells and their mitochondria.

Then, we examined the signal proteins associated with mitochondrial damage that lead to cell apoptosis. It was previously reported that activated p53 (phosphorylated at Ser 15) and p53 levels were important to induce cardiomyocyte mitochondrial damage and finally cell death [37], and p53 levels are usually dependent on the activation of MAPK family proteins such as p-38, ERK and JNK. In our experiment, the phosphorylation levels of p-38, ERK and JNK and p53 in H9C2 cells did not significantly change following the addition of A35, despite the fact that these protein levels were obviously elevated in the doxorubicin-treated group. The levels of downstream targeting protein of p53 such as Bcl-2 did not change in A35-treated cells (Figure 5D).

Annexin V-PI was used to evaluate the apoptosis or necrosis of tumor cells treated with A35, and the results indicated that the apoptotic cells were approximately 90% of total apoptotic and necrotic cells and increased in a dose-dependent manner (Figure 5E). We also observed the final apoptosis event in which PARP was cleaved after A35 treatment for 24 or 48 hours either in K562 or HepG2 cells (Figure 5F). Cleaved and activated caspase-7 that could cleave PARP was also detected after A35 addition. Usually, caspase-7 can be activated by mitochondrial caspase9 or other non-mitochondrial caspases, but we did not observe cleavage and activation of caspase-9, whose activation represents mitochondrial apoptosis. The expression levels of the proteins that promote mitochondrial apoptosis pathway activation, such as Bax and p53 (although p53 is a mutant in K562 cells), were not increased in the presence of A35, and the expression of Bcl-2, a protein that suppresses mitochondrial apoptosis pathway activation, did not change (Figure 5F).

### A35 suppresses tumor cell growth *in vivo* and demonstrates no toxicity in mouse hearts

**Figure 6:**
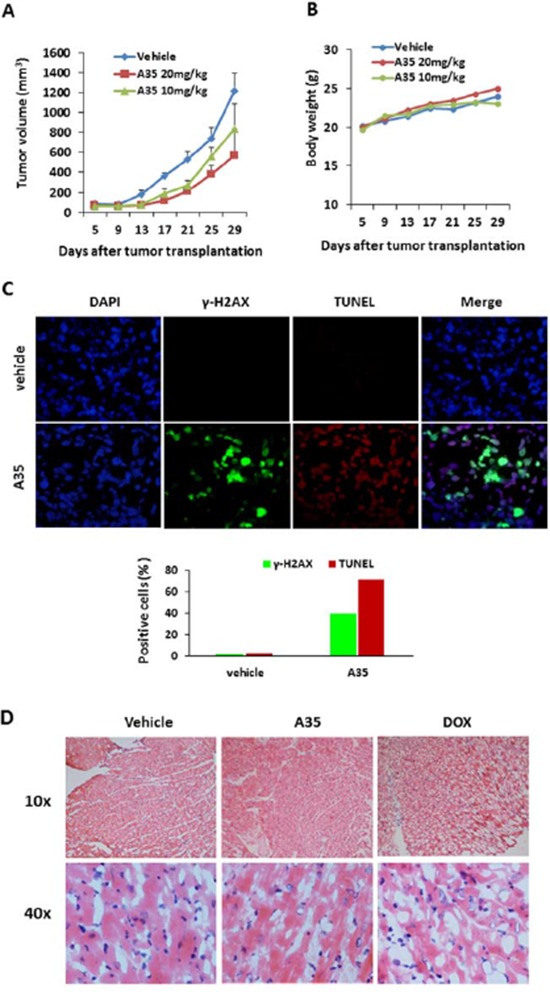

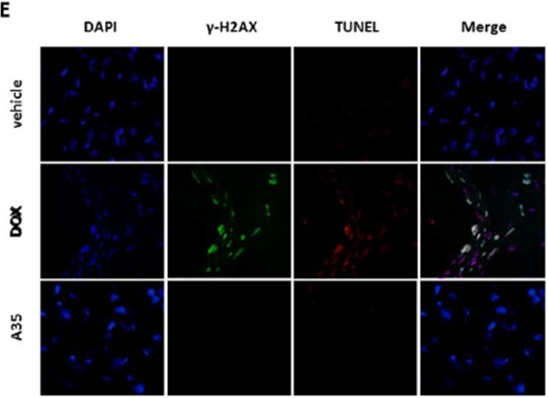
A35 suppresses tumor cell proliferation *in vivo* and has no toxicity in mouse hearts Nude mice (*n* = 5) bearing HepG2 xenografts were administered A35 on day 8 after tumor inoculation and successively administrated A35 for 21 days once per day. Mice were sacrificed when the tumor volume of the control group reached 1000 mm^3^; the tumor loads and hearts were isolated and prepared as frozen cardiac sections. **A.** Tumor volume was measured by calipers twice per week in the indicated days. **B.** Body weights of mice harboring tumors were monitored twice per week in the indicated days. **C.** γ-H2AX immunofluorescence and TUNEL staining of frozen tumor sections from various treatment groups; the total numbers of γ-H2AX-positive nuclei or TUNEL-positive nuclei as a percentage of the total number of nuclei are shown in the bar graph. *n* = 5 mice per group. **D.** H&E staining of frozen cardiac sections from various treatment groups. **E.** γ-H2AX immunofluorescence and TUNEL staining of frozen cardiac sections from various treatment groups.

Next, in a tumor xenograft nude mouse model, we examined A35 anticancer efficacy and its effects on the mouse myocardium. The results indicated that A35 could suppress tumor xenograft proliferation, and at 20 mg/kg the inhibitory rate was approximately 55%, while at 10 mg/kg the inhibitory rate was approximately 35% (Figure 6A). The body weight curves indicated that the animals tolerated well the A35 dosages administered (Figure 6B). When the tumor sizes reached 1000 mm^3^, the mice were sacrificed, and tumors and hearts were excised to be used for further analysis. Tumor tissue was prepared as frozen sections for γ-H2AX detection and for a TUNEL assay to detect apoptosis. The results showed that A35 could significantly induce DNA double breakage, and γ-H2AX-positive cells increased to 40%, and the TUNEL results indicated that A35 could induce tumor cell apoptosis and the apoptotic cells comprised up to approximately 70% of total cells (Figure 6C), indicating an identical action mechanism as for the *in vitro* results.

Cardiac toxicity detection was performed with frozen cardiac tissue sections for H&E staining, γ-H2AX immunofluorescence and the TUNEL assay. H&E results showed that in both the vehicle- and A35-administered groups, the myofibrils all arranged normally, but in the positive control, the DOX-treated group, the myocardial fibers shrank, were distorted and irregularly arranged and the myoplasm significantly lessened (Figure 6D). The TUNEL results corresponded to the H&E results: in the vehicle and A35 groups, apoptotic cells were not observed, but in the DOX group approximately 80% of cells were apoptotic and approximately 40% γ-H2AX-positive cells were observed (Figure 6E).

## DISCUSSION

Cyclizing-berberine A35 is a site 1 and 13 cyclizing berberine. The cyclizing endows this compound with more planar structures that induce intercalation into free DNA, and these aromatic rings enhance the potency of intercalation into topoisomerase [38–40]. This structure is similar to known top2α inhibitor NK314 [22, 23]. After evaluating its effects on top1 and top2α activity, unexpectedly we found that not only could A35 inhibit top2α but it had an effect on top1, indicating that it is a dual topoisomerase inhibitor and has distinct effects on topoisomerases from NK314. Previously, some studies demonstrated that decreased topoisomerase levels are a major mechanism underlying relapse [9] and verified the compensatory effects between top1 and top2, which were also verified in the present study. Additionally, some authors also proposed that a dual targeting topoisomerase might increase overall anti-tumor activity, given that top1 and top2 have overlapping functions in DNA metabolism [41]. Thus, the novel skeleton compound A35 as a dual targeting top1 and top2α inhibitor might have the potency to avoid resistance and produce more powerful anticancer activity.

Given top2α is a more effective target based on its preferential expression in proliferating cells and as the sole enzyme to distort daughter chromosomes, and the stronger effects of A35 on top2α; we focused on studying the inhibitory effects of A35 on top2α. Top2 manipulates DNA topology in an ATP-dependent manner via the mechanism known as the “catalytic cycle”. The interruption of any step in the catalytic process might obstruct enzyme activity. Our results showed that although A35 could intercalate into DNA, it did not disrupt the top2α-DNA interaction. The ATP hydrolysis assay also demonstrated that A35 did not block top2α ATPase activity. Cleavage assays demonstrated that A35 could stabilize the intermediate DNA-top2α complex by exerting its actions both in pre-strand and post-strand cleavage steps and inhibiting religation. Considering all the results above mentioned, Its mechanism is distinct from other known top2α inhibitors, such as etoposide, which only has a very low affinity for intact DNA and only inserts into DNA cleavage sites and inhibits DNA religation [42, 43], and doxorubicin, which at low concentrations (<1 μM) only inhibits DNA religation and at higher concentrations (>10 μM) it interferes with top2 binding to DNA to exert anticancer effects [44]. Other reported inhibitors, such as the quinolone CP-115, the ellipticines, azatoxins, and the natural flavonoid genistein, either strengthen pre-strand or post-strand cleavage to facilitate top-DNA complex formation [45].

The A35 parental core berberine was reported to inhibit top1 [46] and in our lab we did not observe any inhibitory effects on top1 or top2α at concentration up to 80 μM (data not shown), a concentration at which A35 could obviously suppress top1 and top2α activity, indicated a higher topoisomerase inhibitory activity than berberine. Although some studies showed that berberine structural analog 5,6-dihydrocoralyne possessed inhibitory activity against top1 and top2, they did not clarify the effect on top2 isoforms [47, 48]. Berberine and its structural analog trigger mitochondria-dependent apoptosis of cancer cells, and studies also found that mitochondria membrane potential was severely destroyed [27, 49]; meanwhile, p53 was activated, levels of Bax, which can trigger mitochondrial caspase activation, increased and inhibitory protein Bcl2 levels decreased [27, 50, 51]. However, in A35-treated cancer cells, although apoptosis appeared, the mitochondria apoptosis pathway was not activated, which indicated the distinct mechanism from its parental core berberine in apoptosis induction. Usually except mitochondria caspase9, some non-mitochondrial caspases such as caspase-2, caspase-8 and caspase10 also play key role in activating effector caspase-7 [52], thus we speculate A35-induced caspase-7 activation and subsequent apoptosis might be a result of activation of non-mitochondrial caspases. This also arouses our interest and in the following study we will continue working on this issue.

Human top2α and top2β share very similar catalytic activities and are highly conserved, with 78% amino acid identity [53]; many agents targeting top2α also interfere with top2β, such as doxorubicin. However, as the sole enzyme present in heart tissue, the targeting of top2β would disrupt the normal catalytic cycle of top2β and then cause DNA DSB formation and mitochondria destruction, and damage to these organelles would activate the mitochondria apoptosis pathway mediated by p53, which underlies so-called cardiac toxicity [14]. Some reports have also demonstrated that embryonic fibroblasts lacking top2β were better protected against doxorubicin-induced cytotoxicity [54], and top2β deletion mice better prevented doxorubicin-induced cardiomyopathy [5]. Another advantage of only targeting top2α and not targeting top2β is the potential to avoid secondary malignancies. Top2β has been suggested as a major culprit in the development of secondary malignancies [55]. Currently, many novel compounds have also been demonstrated to specifically target top2α, but these data were only from cell-free assays, i.e., compound, extracted topoisomerase enzyme and DNA plasmid directly reacted in buffer. However, validation with cardiomyocytes and animal hearts has not been reported. In our study, not only did we confirm the target in a cell-free assay but also using rat H9C2 cardiomyocytes and nude mice, and we demonstrated that A35 did not induce DNA breakage, mitochondrial injury, apoptosis and p53-mediated mitochondrial apoptosis pathway activation in cardiac cells, which are the main cellular alterations following the targeting of top2β, although in the DOX-treated group the above-mentioned effects were all observed, which further indicated that there were no effects of A35 on top2β at the cellular and animal levels.

In summary, as a novel and DNA intercalative agent, A35 dually inhibits topoisomerase and preferentially and specially targets top2α. It acts as a poison to promote DNA-top complex formation and had no effect on other catalytic steps mediated by top2α. Its distinct mechanisms from other known poisons will be useful for its combination with other topisomerase inhibitors. Although A35 intensively induced cancer cell apoptosis, it did not trigger the apoptosis of cardiac cells and mouse hearts and did not damage the mitochondria either in cancer cells or cardiac cells. Its further exploration might be helpful to overcome top1 and top2 resistant and cardiac toxicity, A35 is a promising topoisomerase anticancer agent and worthy to further develop in future.

## MATERIALS AND METHODS

### Reagents and cells

ICRF-193, VP16 and mAMSA were purchased from Sigma-Aldrich. Anti-γ-H2AX, anti-phospho-p53 (Ser15), anti-p53, anti-phospho-p44/42MAPK (ERK1/2), anti-p44/42MAPK (ERK1/2), anti-phospho-p38 (Thr180/Tyr182), anti-p38, anti-phospho-JNK (Thr183/Tyr185), anti-JNK, anti-caspase3, anti-caspase7, anti-PARP, anti-Bcl2 and anti-Bax antibodies were purchased from Cell Signaling Technology. The anti-β-actin antibody was obtained from Sigma-Aldrich, and peroxidase-conjugated goat anti-mouse or goat anti-rabbit secondary antibodies were purchased from ZSGQ-BIO Company. Antibodies against top1, top2α and top2β were purchased from Abcam. pBR322 DNA and top1 were purchased from BEIJING LIUHE TONG TRADE CO., LTD. Recombinant human top2α was purchased from Topogen. The comet assay kit was obtained from Trevigen.

### Cell lines

Rat myoblasts H9C2 were obtained from the Cell Center of the Institute of Basic Medical Sciences, Chinese Academy of Medical Sciences and Peking Union Medical College. Other cell lines, such as K562, HL60, Raji, Romas, HepG2, Bel7402, Bel7404, HT29 and SMC7721, were either from Cell Center of the Institute of Basic Medical Sciences or from ATCC. K562, HL60, Raji and Romas cells were cultured in 1640 medium with 10% FBS, while H9C2, HepG2, Bel7402, Bel7404, HT29 and SMC7721 were cultured in DMEM with 10% FBS.

### Topoisomerase-mediated DNA relaxation assay

The DNA relaxation assay was based on a procedure described previously [56]. Briefly, 2 μl of 10x reaction buffer with 1 mM ATP (top2α) or without ATP (top1), 0.5 μg of supercoiled pBR322, and 1 unit of top2α (Topogen) or top1 and compound were mixed in a total of 20 μl of reaction buffer. Relaxation was performed at 37°C for 30 min and stopped by the addition of 2.5 μl of stop solution (100 mM EDTA, 0.5% SDS, 50% glycerol, 0.05% bromophenol blue). Electrophoresis was performed in a 1% agarose gel in 0.5x TBE at 4 V/cm for 1.5 hr. DNA bands were stained with the nucleic acid dye EB and photographed with 300 nm UV transillumination. The DNA bands were qualified with software Image J. The percent of relaxed DNA was calculated as: (R-R_0_)/(R_control_-R_0_), where R is the intensity of relaxed DNA incubated with top2α and compound, R_0_ is the intensity of relaxed DNA of pBR322 and R_control_ is the intensity of relaxed DNA incubated with top1. The IC50 was defined as the concentration of A35 that resulted in a 50% reduction of relaxed DNA.

### Top1-mediated DNA cleavage

DNA cleavage assays were performed as described previously [57–59] A 117-bp DNA oligonucleotide from Sangon Biotech encompassing the previously identified top1 cleavage sites identified in the 161-bp fragment from pBluescript SK(−) phagemid DNA was employed. This 117-bp oligonucleotide contains a single 5′-cytosine overhang, which was 3′-end labeled by fill-in reaction with [α-^32^P]-dGTP in reaction 2 buffer (50 mMTris-HCl, pH 8.0, 100 mM MgCl_2_, 50 mM NaCl) with 0.5 units of DNA polymerase I (Klenow fragment, New England BioLabs). Unincorporated ^32^P-dGTP was removed using mini Quick Spin DNA columns (Roche), and the eluate containing the 3′-end-labeled DNA substrate was collected. Approximately 3 nM of radiolabeled DNA substrate was incubated with recombinant top1 (60U) in 20 μl of reaction buffer [10 mM TrisHCl (pH 7.5), 50 mM KCl, 5 mM MgCl_2_, 0.1 mM EDTA, and 15 μg/ml BSA] at 25°C for 30 min in the presence of various drug concentrations. The reactions were terminated by adding SDS (0.5% final concentration) followed by the addition of two volumes of loading dye (80% formamide, 10 mM sodium hydroxide, 1 mM sodium EDTA, 0.25% xylene cyanol, and 0.25% bromophenol blue). Aliquots of each reaction mixture were subjected to 16% denaturing PAGE. Gels were dried and visualized by using a phosphoimager.

### Topoisomerase 1-mediated DNA unwinding assay

To examine the effects of A35 on DNA intercalation, a top1-based assay was carried out according to the literature [20]. In brief, supercoiled plasmid pBR322 was firstly relaxed with recombinant human top1 (4 units) at 37°C for 30 min. Subsequently, A35 or mAMSA was added to the reaction and incubated for a further 30 min at 37°C. Reactions were stopped by the addition of SDS to a final concentration of 1% w/v, and top1 was digested by the addition of proteinase K (50 μg) and incubation for 1 h at 56°C. Samples were resolved on a 1% w/v agarose gel in 0.5x TBE at 4 V/cm for 2 hr and stained with EB. The definition of the percent of relaxed DNA and IC50 was as described in the “Topoisomerase-mediated DNA relaxation assay” section in the Methods.

### Electrophoretic mobility shift assay

The effect of A35 on the binding of top2α to DNA was evaluated using an electrophoretic mobility shift (EMSA) kit (Invitrogen) as previously described [60] according to the manufacturer's protocol. Oligonucleotides containing a strong top2 binding site corresponding to residues 87–126 of the pBR322 plasmid were annealed, and 1 nm was incubated with nuclear extracts (5 μg) from K562 cells in reaction buffer (750 mM KCl, 0.5 mM dithiothreitol, 0.5 mM EDTA, 50 mM Tris, pH 7.4) on ice for 30 min. Then, the samples were electrophoresed on a 5% non-denaturing polyacrylamide gel at 100 V and 4°C in TBE buffer for 1.5 h. DNA was stained with SYBR Green and detected by 300 nm UV transillumination. In A35-treated samples, A35 and nuclear extracts were first incubated for 10 min on ice prior to the addition of oligonucleotide probes, and incubation continued for 30 min on ice. In super shift experiments, an antibody against top2 was first incubated with nuclear extracts for 1 h on ice, and then 1 nm probes were added and incubation continued for 30 min on ice.

### ATPase assay

ATPase activity of top2α was examined by measuring the liberated phosphate of [γ-^32^P]-ATP by thin layer chromatography as previously described [20, 61]. Briefly, top2α (8 units) was incubated in reaction buffer A (Topogen) in the presence of 1.2 μg pBluescript-KS(+) plasmid DNA and the indicated drug for 10 min at room temperature prior to initiating the reaction with the addition of 3 μCi of [γ-^32^P]-ATP (PerkinElmer; 3000 Ci/mmol) and continued to incubate at 37°C. Aliquots (2 μl) were withdrawn at various time points (0, 5 min, 10 min. 15 min, 20 min) and loaded onto pre-washed polyethyleneimine-impregnated cellulose plates (Sigma-Aldrich) and air-dried. Reaction mixtures were resolved by developing plates with freshly prepared NH_4_HCO_3_ (0.4 M). Plates were air-dried and exposed to autoradiographic film. Spots corresponding to free phosphate were excised from the thin layer chromatography plates and quantified using a scintillation counter.

### Top2-mediated DNA cleavage

DNA cleavage assay was performed by using a Top2α Drug Screening Kit (Topogen), 0.2 μg pBR322 DNA plasmid was incubated with top2α or top2β which was obtained and purified as described previously [62] in 20 μl of assay buffer at 37°C for 30 min in the presence or absence of A35 or etoposide. DNA cleavage products were trapped by the addition of 2 μl of 10% SDS and 1.5 μl of 10 mg/ml proteinase K, and then incubation continued for 60 min at 56°C to digest top2α. The samples were mixed with 2.5 μl of loading buffer and subjected to electrophoresis in 1.4% agarose containing 0.5 mg/ml EB at 12 V for 15 hr. All DNA forms were separated and migrated as follows: RLX, SC, LNR, and NC.

### ATP-independent pre-strand passage cleavage assay

Top2α-mediated plasmid DNA cleavage in the absence of nucleotide triphosphate was performed as described with slight modifications [56, 63]. Top2α (8 μnits) was incubated with 0.2 μg of pBR322 with increasing concentrations of A35 or ICRF-193 in 20 μl of reaction buffer. The final buffer contained 0.01 M Tris pH 7.7, 0.05 M NaCl, 0.05 M KCl, 0.1 mM EDTA, 0.005 M MgCl_2_ and 0.03 μg/μl bovine serum albumin. Reactions were incubated for 20 min at 37°C and terminated by addition of 2 μl of 10% SDS, followed by addition of 1.5 μl of 0.25M EDTA and continued to culture for 5 min at 37°C. Then, reactions were digested with 8 μg of proteinase K and incubated for 60 min at 56°C. DNA products were separated on a 1.4% gel containing 0.7 μg/μl ethidium bromide.

### Post-strand passage cleavage assay

A top2α-mediated post-strand passage cleavage assay was performed as described above for pre-strand passage cleavage with the exception that 1 mM AMPPNP (Sigma) was added in the reactions [63].

### ATP-independent DNA religation assay

Top2α-mediated religation of DNA in the absence of ATP was performed as described previously [20]. Top2α (8 units) was incubated with 0.2 μg of pBR322 plasmid DNA in a reaction buffer containing 0.01 M Tris pH 7.7, 0.05 M NaCl, 0.05 M KCl, 0.1 mM EDTA, 0.005 M CaCl_2_ and 0.03 μg/μl of bovine serum albumin for 10 min at 37°C. Immediately after, various concentrations A35 or control were added, followed by the addition of 2 μl of 0.1 M EDTA to the reactions. Then, the reactions were re-initiated by the addition of 0.1 M MgCl_2_ (2 μl) and transferred immediately onto ice. At the indicated time points (15, 30 or 60 seconds), reactions were terminated by the addition of 1% w/v SDS and incubation continued at 37°C for 5 min. The reactions were then incubated with proteinase K (8 μg) for 30 min at 56°C. DNA products were separated on an agarose gel (1.4% w/v) containing ethidium bromide (0.5 μg/μl).

### Comet assay

Top1- or Top2-mediated DNA breakage was measured with a neutral comet assay (Trevigen) for DSB detection or an alkaline comet assay for single-strand break detection as described in the manufacturer's procedures and the literature [64]. The treated cells were embedded in agarose on a slide and subjected to lysis followed by electrophoresis under neutral or alkaline conditions. During electrophoresis, the damaged and fragmented negatively charged DNA migrated away from the nucleus toward the anode. The amount of migrated DNA was a measure of the extent of DNA damage. To detect DNA, the slides were stained with SYBR Gold (Life Technology) staining solution. The slides were examined by fluorescence microscopy (Olympus), and the results were analyzed with the comet analysis software CASP to quantify DNA damage. For each drug concentration, 3 independent assays were conducted in which comet tails were analyzed in a minimum of 50 randomly selected cells in each assay, and parameter reflecting the DNA damage was represented as TM (tail moments, percentage of DNA in tail/tail length) [65].

### TARDIS assay

The TARDIS assay is used to determine cleavable complex formation and has been described in detail [35]. Briefly, cells treated with various concentrations of A35 for 1.5 hr were embedded in agarose on microscope slides and subjected to a lysis procedure that removed the cell membrane and soluble proteins in lysis buffer (1% sarkosyl; 80 mM phosphate, pH6.8; 10 mM EDTA plus protease inhibitors). To remove noncovalently bound nuclear proteins, cells were washed with 1 M NaCl plus protease inhibitors. Then, slides were stained with primary antibody (specific for top1, top2α or top2β, with a dilution of 1:200) and a fluorescein isothiocyanate (FITC)–conjugated secondary antibody. Finally, the slides were mounted with an anti-quenching agent containing DAPI. Images were captured using a fluorescence microscope that separately visualizes green (FITC-stained) and blue (DAPI-stained) fluorescence.

### Small interfering RNA (siRNA)-mediated Gene knockdown

Topoisomerase knockdown experiments were carried out in the same manner as described previously [66]. Small interfering RNAs (siRNAs) were synthesized by Ribo Technology Company using 2′-ACE protection chemistry. The sequences targeting top1, 2α and 2β were, respectively, 5′ GGACUCCAUCAGAUACUAUTT 3′, 5′GGUAUUCCUGUUGUUGAAC 3 'and 5′ AGCCCGAUCCUUUCUUCAUUU 3′.

### Western blot

Whole-cell lysates were used for immunoblotting as described previously [67].

### Measurement of mitochondrial membrane potential

5,5′,6,6′-tetrachloro-1,1′,3,3′-tetraethylbenzimidazolocar bocyanine iodide (JC-1) dye (sigma) exhibits potential-dependent accumulation in the mitochondria, and JC-1 selectively enters the mitochondria and spontaneously forms complexes known as J-aggregates. If the membrane potential is disturbed, the dye remains in the monomeric form, emitting only green fluorescence. Thus, this dye was employed to detect changes in the mitochondrial membrane potential (ΔΨm). JC-1 dye was added to the culture medium at 10 μg/ml and incubated for 15 min at 37°C. After mounting on the slides, the cells were immediately examined under a fluorescence microscope (Olympus).

### *In vivo* antitumor activity

The *in vivo* efficacy of A35 was evaluated with HepG2 xenografts in nude mouse (purchased from Experimental Animals, Chinese Academy of Medical Sciences & Peking Union Medical College). First, 1 × 10^7^ HepG2 cells suspended in 200 μl of PBS were inoculated s.c. in the right armpits of nude mice. After 3 weeks, the tumors were removed from the nude mice and dissected aseptically in sterile saline. Pieces of tumor tissue (2 mm^3^ in size) were then transplanted into the right armpits of nude mice with a trocar. Tumor-bearing mice were randomly divided into 3 groups (*n* = 5) when the tumor size was about 100 mm^3^. A35 (10 or 20 mg/kg) was administered by intraperitoneal injection once a day until the mice were sacrificed. Tumor size was measured every 3 days, and tumor volume was determined as length × width^2^/2. Mice were killed when the tumor volumes of the control group reached 1000 mm^3^; the tumor loads and hearts were isolated and used in further assays.

### H & E staining, detection of apoptosis and immunofluorescence of tissue sections

Hearts from A35-administered mice were excised and preserved in liquid nitrogen until frozen heart sections were produced. In the cardiotoxicity positive control group, DOX was administered by intraperitoneal injection at 0.5 mg/kg once every two days. H&E staining was performed as described in [68]. Slides were incubated with 0.5% Triton for 20 min and blocked with FBS at 37°C for 30 min, and then γ-H2AX antibody conjugated fluorescein isothiocyanate (FITC) (BD) was added overnight at 4°C, followed by the addition of TUNEL reaction buffer (Roche Applied Science) for 10 min. The nuclei were stained with DAPI (Sigma) before the slides were sealed and examined with a fluorescence microscope (Olympus). The γ-H2AX-positive cells and cells with apoptotic nuclei were counted at 400× magnification to obtain the total nuclei per section. Five sections from each mouse were counted and averaged.
